# The Establishment of a Tobramycin-Responsive Whole-Cell Micro-Biosensor Based on an Artificial Ribozyme Switch

**DOI:** 10.3390/life13071553

**Published:** 2023-07-13

**Authors:** Zhenzhen Wang, Jiawen Cheng, Guimin Dai, Xiaoqi Sun, Xueli Yin, Yuanyuan Zhang

**Affiliations:** 1 School of Life Science, Anhui Medical University, Hefei 230032, China; 2 School of Basic Medical Sciences, Anhui Medical University, Hefei 230032, China

**Keywords:** tobramycin, tobramycin-dependent hammerhead ribozyme-based aptazyme (tob-HHAz), whole-cell microbial sensor

## Abstract

In this study, a tobramycin concentration-dependent whole-cell micro-biosensor (tob-HHAz) was constructed by fusing a tobramycin aptamer with a hammerhead ribozyme (HHR) from *Schistosoma mansoni*. The biosensor was obtained by integrating all the modules into one complete RNA sequence, which was easily introduced into *E. coli* without suffering from harsh external environments. Three independent tobramycin-sensitive RNA structures were identified via high-throughput screening in vivo and were further verified in vitro to undergo the desired self-cleavage reaction. The computation prediction of the RNA structure was performed to help analyze the mechanisms of various conformations by performing a qualitative and rapid detection of tobramycin in practical samples; two sensors exhibited high responsiveness to spiked milk, with a detection limit of around 40 nM, which is below the EU’s antibiotic maximum residual level. One of the structures provides a linear range from 30 to 650 nM with a minimum detection limit of 30 nM and showed relatively good selectivity in spiked urine. This study is the first in which in vivo screening was combined with computation analysis to optimize the pivotal structure of sensors. This strategy enables researchers to use artificial ribozyme-based biosensors not only for antibiotic detection but also as a generally applicable method for the further detection of substances in living cells.

## 1. Introduction

Tobramycin (tob), as an important category of aminoglycoside antibiotics (AAs), has been widely utilized in animal husbandry and the medical field due to its broad-spectrum antibacterial activity. Nevertheless, tobramycin residues are prevalent in both food and the environment due to their persistent accumulation within the ecological system. This poses a high risk to human health, leading to potential complications such as nephrotoxicity and inner ear toxicity [1,2]. In addition, the excessive use of AAs results in increased antibiotic resistance in bacteria, which is becoming a global public health problem [3]. Therefore, a convenient method for tobramycin detection is important for evaluating their environmental impact and regulation [4,5].

To date, the residue levels of AAs in samples are mainly analyzed using an enzyme-linked immunosorbent assay (ELISA) [6], chromatographic methods (LC/LMC) [7,8], capillary electrophoresis (CE) [9], and capillary electrophoresis–mass spectrometry (CE-MS) [8,10], with high accuracy and sensitivity. However, there are some inherent disadvantages to these techniques, such as tedious sample pretreatment and sophisticated instrumentation operation [5]. Thus, the development of an inexpensive and convenient detection method for animal-derived foods or the human body is still of high research interest.

The application of biosensors, through which different targets can be quantitatively or qualitatively determined by producing a physically detectable signal, has opened the door to developing a new method for more inexpensive and convenient detection of AA residues [11,12]. Among the different analytical biosensors, aptamer-based biosensors (aptasensor) [13,14] have high precision and programmability. By using aptamer as the biological recognition element, the RNA structure is employed as a signal transduction domain for the detection of various structures, from ions to small organic molecules, proteins, cells, and even the entire virus particle [15,16,17,18]. Furthermore, the variable modular construction of aptamers allows for enough flexibility to modify the design, thus satisfying a wide range of applications [19].

Ribozyme is a class of small molecular RNA with a self-cleavage function. By fusing catalytic motifs to an aptamer domain, the RNA integrates sensor and regulatory functions into one scaffold, thereby frequently being utilized for constructing the artificial allosteric ribozyme called aptazyme [20,21]. 

By integrating aptazyme with either 5′ or 3′UTR of a reporter gene in a well-designed and tested construct, it can be harnessed to create a biosensor to detect specific small molecules in prokaryotes and eukaryotes [22]. A series of allosteric ribozymes that are sensitive to various effector molecules, such as ATP, TPP, second messenger cGMP/cAMP, theophylline, and the FMN (flavin mononucleotide), have been created. These engineered allosteric ribozymes have been used in the prototype multiplex biosensor array [23].

In principle, this enables the production of various allosteric ribozymes that are selective to different small molecules. However, allosteric ribozyme screening is time-consuming and requires complex technical equipment, and the selected allosteric ribozymes in vitro may lose their function when transferred to the cellular system [24]. Thus, an alternative strategy is the generation of random libraries, followed by the high-throughput analysis of ligand-dependent genetic switches in cellular systems [25]. Random libraries of ribosome switches have been successfully screened in bacteria or yeast using fluorescently activated cell-sorting procedures with automated devices [26]. Furthermore, in vivo selection schemes based on bacterial anti-selection markers instead of the traditional report gene have been used for generating artificial ribosome switches [27,28].

However, this screening based on random libraries is still unpredictable and requires arduous work. The primary prediction of the secondary structure of allosteric ribozyme RNAs using relevant bioinformatic tools is helpful to further understand the characteristics of the allosteric ribozymes and expedites the rational construction of ligand concentration-dependent biosensors.

Aptazyme-based biosensors can compensate for the defects of traditional methods using probe labeling [29,30], which allows for their extensive application to detect antibiotics in food or medical samples. In addition, complex biological concepts that are linked with convenience readouts make testing easier, even without sophisticated instruments [31]. 

Most aptazyme-based biosensors have been realized in whole-cell expression (WCE) [30], which enables researchers to program microbes for the production of reporters that are easily detected and easily and inexpensively produced on a large scale as living sensors.

This study is the first to report a ribozyme switch that can be used for tobramycin detection. A tobramycin concentration-dependent micro-biosensor (tob-HHAz) was obtained by integrating various modules into a common, complete RNA sequence, which was then introduced into *Escherichia coli* to establish a whole-cell microbial sensor system. 

This is the first time that in vivo screening is combined with computation analysis to optimize the pivotal structure of sensors. Whole-cell micro-biosensors were well positioned to address the important goal of antibiotic detection. For features of the aptamer variable region, the sensor also provides a generally applicable method for the further detection of substances, ranging from small antibiotic molecules to heavy metal ions, amino acids, proteins, etc., in living cells, which highlights the great significance of applying these biosensors in assays that are performed for food safety control, environmental monitoring, and medical detection. 

## 2. Materials and Methods

All the initial plasmids and bacteria strains were stored in the laboratory, and the plasmids were constructed by using standard molecular techniques. Primer and template sequences are shown in Table S1. All oligonucleotides were synthesized by Sangon, Shanghai, China. Cloning enzymes, including restriction enzymes and T4 DNA ligase, were obtained from Takara or NEB Company. Plasmids were isolated using a plasmid kit (Miniprep Kit, Qiagen, Hilden, Germany), and commonly used biochemical reagents were purchased from Sinopharm.

### 2.1. Bacterial Vitality Test

In order to investigate the expression of eGFP in *Escherichia coli* and lay a foundation for the subsequent screening of ribozyme switches that can regulate *Escherichia coli* expression based on small molecules, pET-16b was used as a negative control, and the artificially constructed plasmid was transformed into *E. coli.* Strain BL21 (DE3) and the single clone were picked out and transferred into 96-well plates at 3 °C, 200 rpm, to develop culture until the optical density value at 600 nm (OD600) reached 0.6. IPTG (1.0 mM) was added to the medium (with various concentrations or without antibiotics) and agitated at 200 rpm for 8–12 h. Fluorescence and OD600 measurements were carried out every two hours at room temperature on a microplate reader (TECAN Spark, Männedorf, Switzerland), with an excitation wavelength of 480 nm and a 525 nm emission filter. All experiments were carried out in triplicate. The results were obtained by using a negative sample (pET-16b) to remove the background cellular fluorescence and minimize the toxicity effects of the antibiotic. The background-corrected fluorescence data were further normalized using OD600. The optimal cultivation time was determined at 10 h. This method can be extended by monitoring bacterial vitality with the use of different antibiotic molecules. For each sample, the fluorescence of the induced cells (+ antibiotic) was normalized with the fluorescence of the uninduced cells.

### 2.2. Library Construction

The eGFP gene was used for PCR from pEGFP-N1 and was inserted into pET-16b by digesting the plasmids with *BamHI* and *NdeI*. The plasmids that harbored HHR were engineered by inserting sequences into the plasmid pET-eGFP via overlap PCR method. Three random nucleotide (NNN) bridges were designed for library construction, which were flanked at the aptamer and the HHR domain. 

### 2.3. In Vivo Screening of Aptazyme Libraries

The constructed plasmids were transformed into *E. coli* BL21 (DE3) for in vivo screening. *E. coli* was grown in an LB medium supplemented with 100 μg/mL ampicillin.

Negative screening: Single clones of the transformed HHAz pool were picked out and transferred into 96-deep well plates for incubation with 1 mM, IPTG for 8 h (37 °C; 200 rpm; pET-16b and pET-EGFP as negative or positive control). Fluorescence and OD600 measurements, as well as data processing, were carried out similarly to bacterial vitality and fluorescence expression tests. Then, the clones with high background fluorescence were removed, and the rest clones were transferred into two plates with or without tobramycin (3 μM). 

Positive screening: The bacteria were cultured under the same conditions. The clones with improved relative fluorescence intensity were chosen for the next round of screening. After three rounds of rotation screening, 15 candidates with the best relative fluorescence intensity were obtained for subsequent sequence analysis, 4 of which were used due to their correct sequence. 

### 2.4. In Vitro Self-Cleavage Reactions

In vitro transcription was carried out using an RNA MEGA script™ T7 kit (Thermo Fisher, Waltham, MA, USA), and the protocol was conducted according to the directions of the kit. The transcript RNA sample was collected, and the reaction was immediately stopped by adding a denaturing quench buffer (80% methanamide, 20 mM EDTA, with 0.05% xylene cyanol, 1 mM Tris, pH 7.5). Then, in vitro cleavage kinetic assays were initiated by incubating the RNA sample (5 μL) with the reaction buffer (25 μL of 100 mM MgCl_2_, 100 mM NaCl, 250 mM Tris-HCl, and pH 7.5) at 37 °C. The aliquots (3 μL) prepared at different time points were quenched with the buffer. The resulting gels were used to measure the observed band intensities and thus investigate the progress of the initiated cleavage reaction over time. 

Dose-dependent verification: The aliquots of RNA (3 μL) were prepared by adding different concentrations of tobramycin from 0 to 3 µM and quenched with 6 μL of the buffer after 30 min incubation. The reactions were then fractionated using 7% PAGE. After electrophoresis, the gel was stained with GelRed for 15 min and then photographed using the Gel imaging system (Bio-Rad, Hercules, CA, USA).

### 2.5. Tobramycin Detection in Milk and Urine

Preparation of detection tubes: The bacteria preserved at −80 °C were transferred to the LB medium supplemented with 100 μg/mL ampicillin and were agitated at 200 rpm and 37 °C until the OD600 value reached 0.6 (exponential phase). Then, 1 mM of IPTG was added for RNA transcription and induced at 200 rpm, 37 °C, for 2.5 h. Then, 1 mL samples of the activated bacteria were distributed into 1.5 mL tubes and centrifuged for 3 min (4000 rpm). The bacteria pellet was preserved at 4 °C for at least 2 days after the removal of the LB medium supernatant.

Pretreatment of milk and urine samples: Milk was treated with 6 times the volume of 20% acetic acid and left standing for 1 h at 4 °C. After centrifugation for 10 min (4 °C, 8000 rpm), the supernatant was filtered using a 0.22 μm filter membrane to remove lipids. The pH was adjusted to 7.0 with 1 M of sodium hydroxide and then diluted 10 times with a PBS (pH 7.4) buffer solution. The urine samples were treated using a filter (0.22 μm), and they were then directly used for subsequent detection. Ten aliquots of samples with varying concentrations of tob (0–1000 nM) were prepared by spiking different amounts of the standard solution of tob. To further determine the minimum detection concentration of the ligand, the lower concentration (<100 nM) of tob detection was used in the follow-up experiment. All samples were tested in triplicate.

Tob detection: First, 300 μL of the treated milk or urine samples were added to detection tubes. Then, 200 μL of suspension samples were transferred into a 96-well blackboard and were agitated at 200 rpm, 37 °C, for 3 h. Fluorescence measurements and data processing were carried out similarly to the previous tests.

## 3. Results

### 3.1. The Optimal Design of Anti-SD Sequence

Based on Wieland’s research [32], an artificial self-cleaving hammerhead ribozyme (HHR) N79 from *Schistosoma mansoni* [33] with anti-SD (Shine−Dalgarno) sequences was inserted into 5′UTR of mRNAs. The SD-blocked structure inhibited ribosome loading or scanning, thereby downregulating gene expression at the transitional level. The strong auto-catalytic activity of HHR facilitated the release of anti-SD sequence and continuation of translation, and thus the “turn-on” function was achieved for the reporter’s gene expression downstream. 

**Figure 1:**
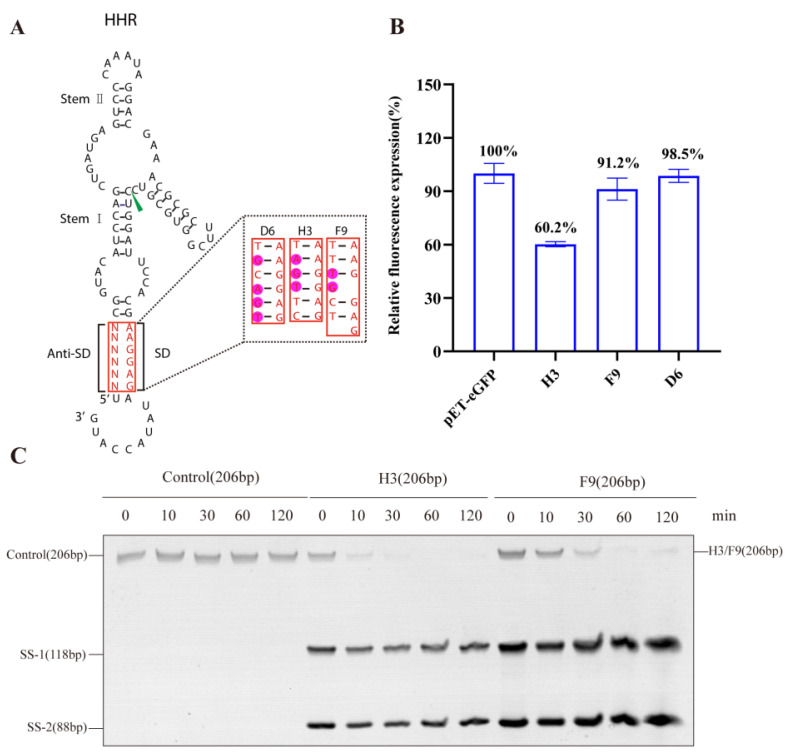
Anti-SD sequences screening of HHR: (**A**) three optimized candidates with various anti-SD sequences; (**B**) fluorescence recovery capability of three optimized candidates. Error bars represent the standard deviation of at least three individual experiments. Fluorescence data were normalized as percentage values with respect to the wild-type sample considered as 100%; (**C**) in vitro cleavage of ribozymes H3 and F9. The sample RNA self-cleavage reaction was stopped by adding denaturing quench buffer at 0, 10, 30, 60, and 120 min, respectively. Samples were analyzed using 7% denaturing PAGE. Full-length HHR (206 bp) with mutant splice site was used as a negative control. The product of HHR after cleavage showed ss-1/ss-2 fragments.

However, the tightly blocked SD design showed weak eGFP fluorescence recovery performance under the IPTG-induced condition, which was inconsistent with the results reported in the literature [32]. We found that the design with −30.50 kcal/mol minimal free energy led to rigid construction, which made its effective release difficult. Therefore, we modified the structure by randomizing the 6nt anti-SD RNA sequence to perform an in vivo screening of *E. coli.* Three candidates were obtained for their best fluorescence intensity recovery performance (Figure 1A,B). Both F9 and D6 candidates restored eGFP expression to the level of positive control to a high degree. Since D6, with 4nt non-complementary sequence to the SD, showed a high initial fluorescence level even without IPTG, H3 and F9 were utilized for the primary verification of HHR construction in the subsequent experiments.

Further in vitro cleavage assays of H3 and F9 were carried out at low Mg^2+^ concentrations (200 mM), and the reactions were quenched with the stopping buffer after defined time points. Full-length HHR with mutant splice sites was used as a negative control. The products were analyzed using urea–polyacrylamide gel electrophoresis (Urea–PAGE) (Figure 1C). Short-sequence (SS) fragments were observed at 0 min, while the full-length HHR was completely digested only 30 min after transcription. H3 and F9 showed strong self-cleaving activity and therefore pave the way for the subsequent construction of systems that employ allosterically regulated ribozymes. 

### 3.2. In Vivo Screening of Tobramycin Dose-Dependent Sensor 

**Figure 2:**
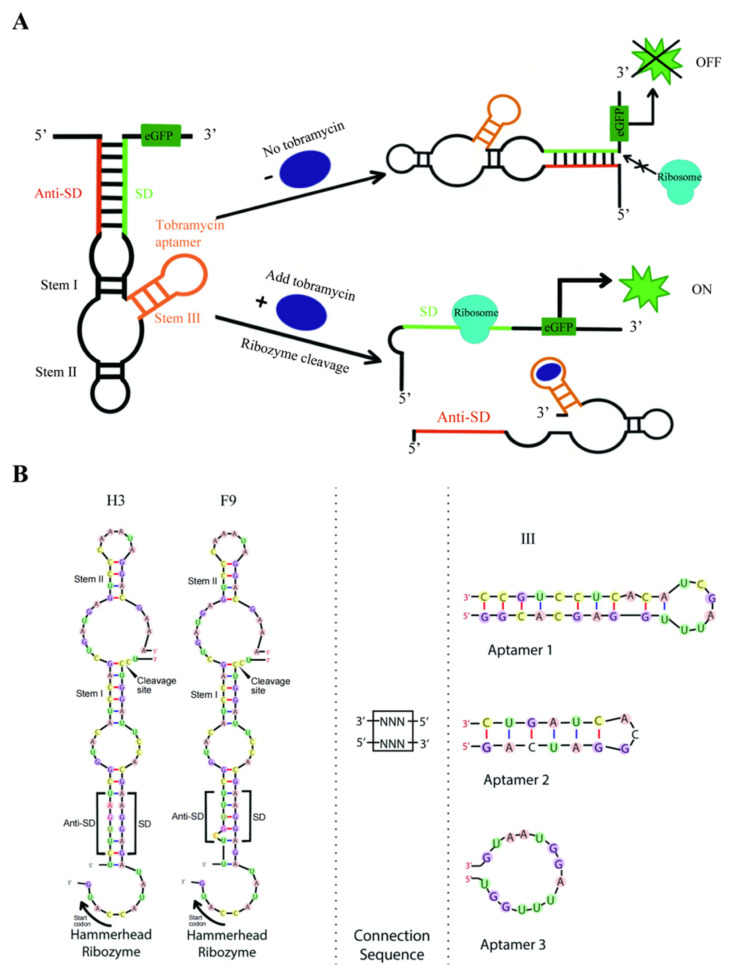
(**A**) Schematic diagram of the strategy of tobramycin dose-dependent sensor. The formation of the aptamer domain of aptazyme resulted in structural rearrangement, which inhibited the catalytic conformation of the ribozyme. In contrast, ligand binding to the aptamer triggered the liberation of the SD sequence and gene expression, thus achieving a “signal-on” fluorescence change process in cells; (**B**) schematic illustration of the secondary library design based on the optimized HHR and tobramycin aptamer candidates. The bridge domain carried a total of three pairs of random sequence nucleotides.

We further engineered tobramycin-dependent hammerhead aptazymes (tob-HHAz) to regulate gene expression by combining the full-length H3/F9 sequence onto the three tested aptamers, namely A1 [34], A2 [35], and A3 [36] (Figure 2B). The formation of the aptamer domain resulted in structural rearrangement, which inhibited the catalytically active conformation of the ribozyme. In contrast, the ligand binding to the aptamers triggered the liberation of the RBS sequence, thus achieving a “signal-on” fluorescence change process in cells (Figure 2A).

Improving the performance of aptazyme-based genetic switches is an important step toward a more widespread application of post-transcriptional gene regulators. Stem III of *Schistosoma mansoni* HHR proved to be a promising platform for influencing the integrity of the catalytic core [37]. Thus, it might be beneficial to keep the domain of self-cleavage sequence as native as possible [23]. In order to retain the essential tertiary contacts for fast cleavage kinetics as well as enable the regulation of the activity of the HHR, optimizing the connecting sequence that communicates ligand binding to the catalytic core is essential [38]. Therefore, the tobramycin aptamer was attached to the stem III of an optimized HHR via a connection sequence of six randomized nucleotides. The corresponding plasmids were then transformed into *E. coli* cells to construct a bacterial library with 2 × 10^5^ single colonies, for about 8 times the capacity of random sequence (4^6^ × 6 members theoretically, with six randomized nucleotides for connection sequence, three tobramycin aptamer candidates, and two optimized HHR structures resulting in six different combinations), which accounted for a greater than 95% coverage of total sequence space. As a high concentration of tobramycin has a strong inhibitory effect on *E. coli* proliferation, 3 µM was determined as the proper concentration of tobramycin for screening using the bacterial growth curve method (Supplementary Figure S1). 

**Figure 3:**
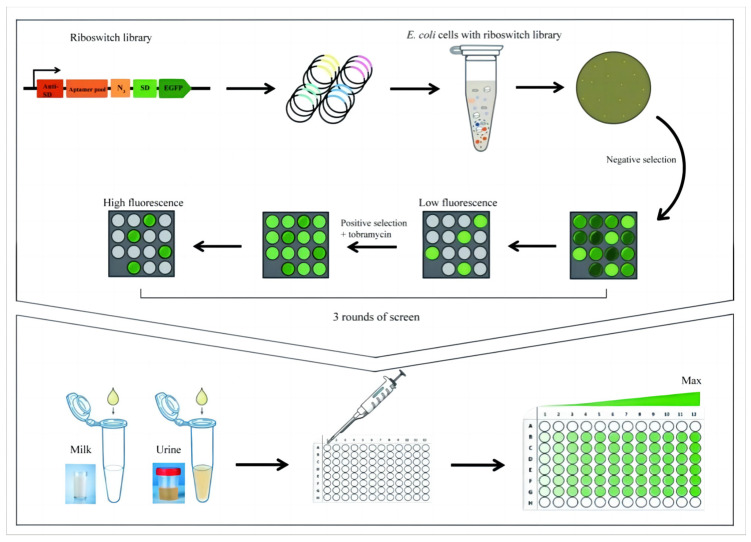
Schematic diagram of the screening of ribozyme-based tobramycin-responsive whole-cell micro-biosensor.

**Figure 4:**
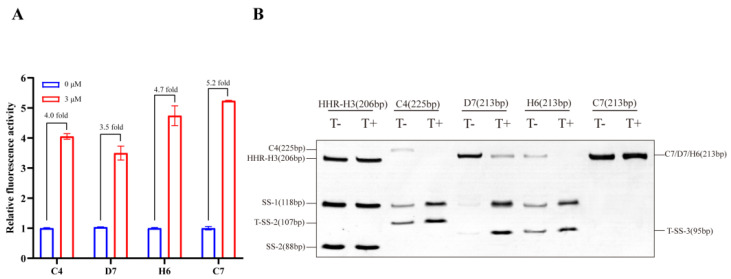
Screening of tobramycin dose-dependent sensor: (**A**) tob-HHAz C4, D7, H6, and C7 were available and exhibited a significant positive fluorescence response to tob. Fluorescence intensity of C4, D7, H6, and C7 in the presence (blue) and absence (red) of 3 μM tob was detected using a microplate reader. Each measurement was repeated in triplicate, and values of relative fluorescent expression were normalized with 0 μM of tobramycin; (**B**) denatured urea–PAGE assay of tob-HHAz self-cleavage reaction in vitro. Cleavage of ribozymes was induced with or without 3 μM tob. The HHR-H3 sample clone lacking the tob aptamer was used as a negative control.

Screening for tobramycin-dependent fluorescence changes in eGFP was performed in a two-step process (Figure 3). First, negative selection was carried out to rule out the cells with high background fluorescence in the absence of ligands. However, tob-HHAz showed higher fluorescence than that of negative controls (pET-16b) even without tobramycin, which may be due to the structure leaking of HHR (confirmed in subsequent in vitro cleavage experiments, as shown in Figure 4B). Therefore, we appropriately raised the background fluorescence threshold, thus avoiding the early elimination of potential screening targets. In the first screening step, only 1.5% of the clones (3000 of 2 × 10^5^ transformants) met the requirement with lower eGFP background in the absence of tobramycin. For the second step, 3000 individual fluorescent colonies were screened using 96-well deep-well plates with 3 µM of tobramycin or without ligands. Plasmids were isolated from individual candidates with the upregulation of eGFP expression and transferred into *E. coli*, after which the eGFP measurement was repeated. Three rounds of rotation screening were performed to remove unstable expressed colons. In total, 15 candidates were identified, which exhibited a significant positive fluorescence response to tob. The sequence analysis of these clones yielded four individual candidates, which displayed strong induction of eGFP with tobramycin (Figure 4A). The bacterial clones D7, C7, and H6 harboring the A2 aptamer sequence afforded 3.5-, 4.7-, and 5.2-fold improvement in fluorescence, respectively. The C4 clone, containing the A1 aptamer sequence, increased 4.0 fold in the presence of 3 µM of tobramycin. There were no efficient A3 aptamer-derived candidates available, which possibly led to unverified splicing conformation in the presence of tobramycin.

### 3.3. In Vitro Transcription and Cleavage of Tob-HHAz Sensor

In order to evaluate whether the changes in eGFP expression observed in vivo resulted from tob-dependent changes in ribozyme activity, the in vitro transcription of the tob-HHAz clones was performed at a low Mg^2+^ concentration for 10 min, and the full-length precursor RNAs were purified via denaturing polyacrylamide gel electrophoresis (PAGE). The results (Figure 4B) show that the HHR-H3 sample clone (negative control) lacking the tob aptamer did not display any changes in response to 3 µM tob. Compared with D7, C4 and H6 exhibited more efficient cleavage rates in response to the ligand, which is in accordance with the in vivo data, indicating their involvement in ligand-induced structural change. Although C7 exhibited the greatest improvement in the fluorescence activation ratio during in vivo screening, it did not display any detectable cleavage fragments. This finding could hint at possible mechanisms in C7 that cause the inhibiting arms to be improperly folded in the presence of the ligand.

These results suggest that altering the sequence variants between the aptamer and ribozyme has a dramatic effect on the function and dynamic range of synthetic tob-HHRAz. Therefore, it is essential to gain insights into their mechanisms of action, which were further investigated.

### 3.4. Tobramycin Concentration-Dependent and Specificity Verification of the Tob-HHAz Sensor 

**Figure 5:**
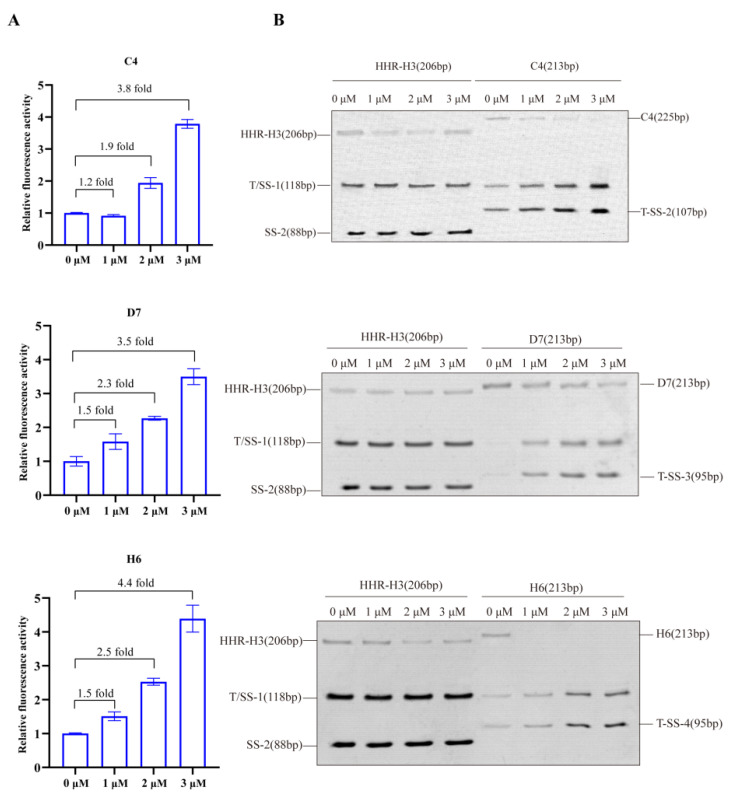
Tobramycin concentration-dependent verification of tob-HHRz sensor: (**A**) the results of in vivo reporter gene assays of C4, D7, and H6 demonstrate the dose–response upregulation of eGFP expression in the presence of tob from 0 to 3 µM (0, 1, 2, and 3 μM); (**B**) in vitro cleavage was induced using tob at various concentrations.

The results of the reporter gene assays of C4, D7, and H6 demonstrate the dose–response improvement in eGFP expression in the presence of tob from 0 to 3 µM, as shown in Figure 5A. The response was indeed performed through the aptamer-based recognition of the ligand. 

The in vitro cleavage analysis of the RNA transcript was used for further verification of the auto-catalysis conformation (Figure 5B). The results were in accordance with the proposed theory and proved the ability of this ribozyme switch system to conditionally improve target gene expression in response to ligand input. Hence, the response was analyzed with the aptamer-based recognition of the ligand.

**Figure 6:**
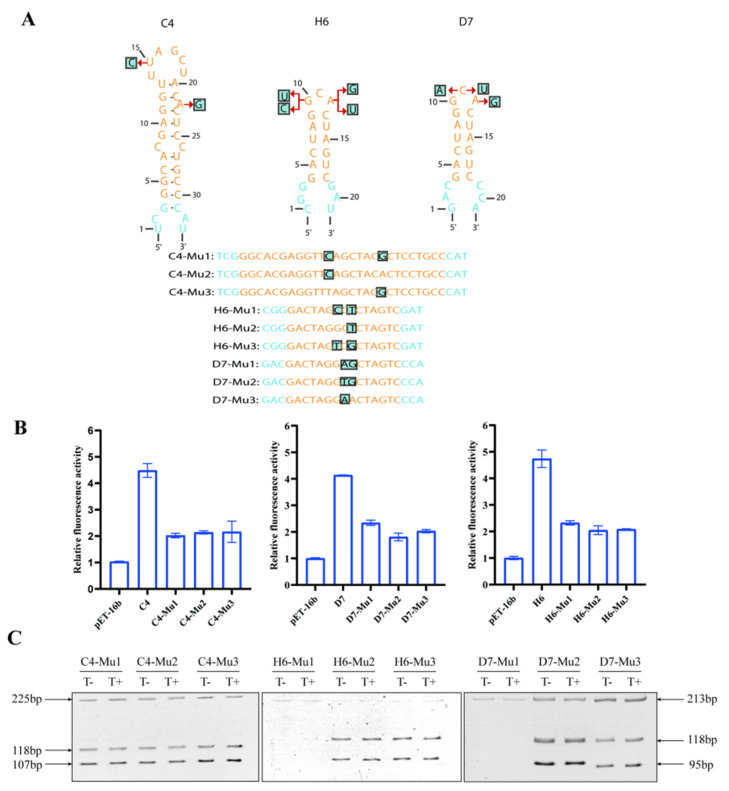
Specificity verification of the tob-HHAz sensors: (**A**) schematic diagram of point mutations on aptamer sequence (yellow). One or two mutants were introduced in the aptamer regions marked with blue boxes. Nucleotides with blue color were flanked using the aptamer’s existing bridge sequence; (**B**) fluorescence expression of various tob-HHAz mutants. eGFP expression was induced with 3 µM of tobramycin or without tobramycin. The data were normalized using the fluorescence expression of negative control; (**C**) the in vitro cleavage analysis of mutant variants.

Next, we carried out the analysis of controls in order to confirm that the switches indeed operate via the tob-dependent initiation of translation. Thus, a series of point mutations were introduced into the aptamer (Figure 6A). If the proposed mechanism of self-cleavage necessary for tob-specific induction was present, an inactivated variant of the aptamer should not display gene expression variations at all.

As expected, the case in which mutant variants were added with 3 µM tob showed weak gene expression improvement compared with its parental tob-HHRAz (Figure 6B), demonstrating that the conserved aptamer sequence is necessary for effective ribozyme cleavage. However, compared with the negative control pET-16b, the fluorescence intensity of the mutant was doubled in the presence of 3 µM tob. The in vitro cleavage analysis of mutant variants verified that the variation in gene expression could only be triggered by strong ligand affinity to its aptamer, but it was difficult to completely shut down the ribozyme’s self-cleavage action through the subtle mutation of the aptamer (Figure 6C). 

### 3.5. The Prediction and Analysis of the Secondary RNA Structure of the Tob-HHAz Sensor

Both in vivo and in vitro experiments demonstrated the significant dose-dependent characteristics and high ligand specificity of tob-HHAz, which are essential traits in sensors. However, the random bridge sequences between HHR and the aptamer pointed to the uncertainty of their catalytic cleavage efficiency. 

Thus, we conducted an M-fold analysis to gain further insight into a possible switching mechanism. Vienna RNA package 2.0 was used for secondary structure prediction and dynamic analysis, as well as the visual analysis of the base pairing probability. 

**Figure 7:**
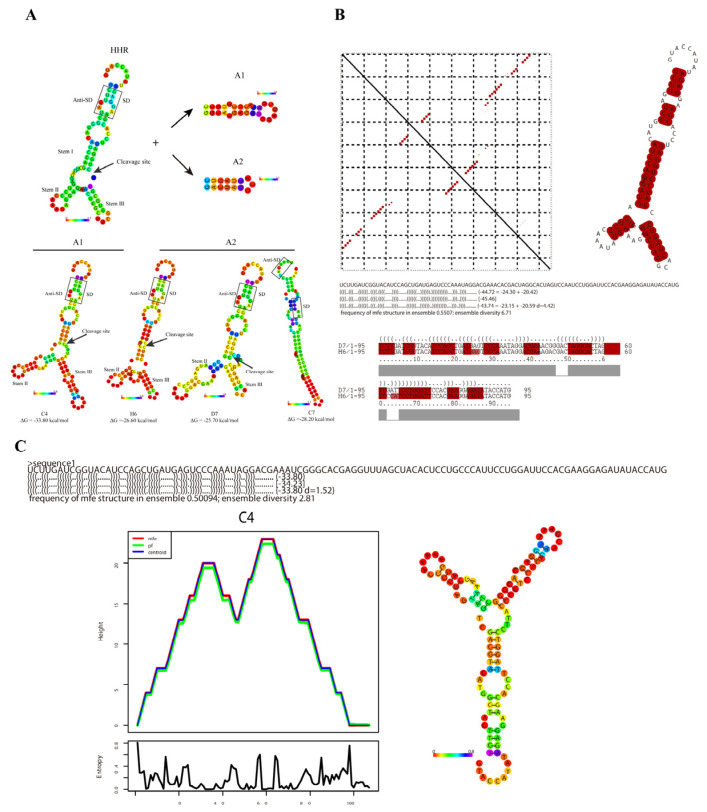
Prediction and analysis of RNA secondary structure of tob-HHAz sensor using Vienna package 2.0: (**A**) Predicted secondary structure of tob-HHAz (color indicates positional entropy). A1, A2 show different aptamer sequences of tobramycin respectively; (**B**) analysis of the base pairing probability and consensus structure on H6 and D7. The probability of the base pairing is proportional to the size of each point, which is shown in the upper triangular matrix. The lower triangular matrix represents the optimal structure resulting from the prediction using minimum free energy. The dot plot distribution shows almost identical morphology in both triangular matrices, suggesting that the construction of H6 and D7 is highly consistent with the predicted results. The conservative sequences shared by H6 and C4 are marked in red color on the right side; (**C**) analysis of the folding dynamics of the C4 sequence and its core structure. The right panel shows a mountain plot of the minimum free energy structure, centroid structure, and partition function structure. The height (y-axis) of the mountain plot indicates the number of base pairs enclosing a sequence position, which is indicated in the x-axis. PE indicates the partition function structure. Centroid indicates the centroid structure. The secondary structure of C4 is located on the right side of the mountain plot.

Firstly, the minimum free energy (MFE) algorithm based on the secondary structure was employed to predict the features of C4, D7, and H6 (Figure 7A). The results show that both C4 and H6 maintained their original secondary structure involving stems I and II of the HHR, which allow for the exposure of the original cleavage site without the influence of base alteration. In addition, the HHR catalytic core, which is crucial for the activation of ribozyme cleavage, maintained its original structure as long as possible. Furthermore, because there was no cleavage in the in vitro experiment, the MFE secondary structure of C7 was analyzed. Interestingly, the catalytic core of C7 was completely blocked without any sequence exposure, confirming the results of the previous in vitro experiment. 

In addition, the RNAfold software was used for further analysis of the folding dynamics of the core structure of the tob-HHRAz RNA. Figure 7C shows the mountain plot of the minimum free energy structure, centroid structure, and partition function structure of the C4 sequence. The close correlation of these curves indicates the fact that C4 with the lowest MFE value had the most stable and conservative construction, compared with D7 or H6. This result is in agreement with the results of the previous experiments.

It has long been known that the accuracy of thermodynamic structure predictions for individual sequences is rather limited. Therefore, analyzing the consensus structure of several related RNA construction sequences would strongly improve the accuracy of the prediction [39].

Since H6 and D7 share the common A2 aptamer, we further analyzed the base pairing probability and consensus structure sequences of them (Figure 7B). Each element in the dot plot matrix represents one base pair. The probability of base pairing is proportional to the size of each point, which is shown in the upper triangular matrix. The lower triangular matrix represents the optimal structure resulting from the prediction with minimum free energy. The dot plot distribution showed almost identical morphology in both triangular matrices, which suggests that the construction of H6 and D7 is highly consistent with the predicted results. The conservative sequences shared by H6 and C4 are marked with red color on the right side, which provides additional support for the accuracy of the conclusion.

These results further support the previous functional verification results of the different aspects of the secondary structure prediction of the sensor. The structures of C4 and H6 were better in terms of the changes in fluorescence regulation in vivo and the efficiency of cleavage in vitro. Therefore, C4 and H6 were used for the subsequent detection of tob in real samples.

### 3.6. Specificity of Tob-HHAz Sensor to the Aminoglycoside Antibiotics 

Specificity is probably the most important feature for the performance evaluation of biosensors. In order to further assess the specificity of the tob-HHAz, several aminoglycoside antibiotics, namely neomycin, paramycin, amikacin, and gentamicin, which have similar antibiotic mechanisms to tobramycin, were analyzed under the same conditions. The efficient concentrations were determined by first generating the cell growth curves (Figure S2). 

**Figure 8:**
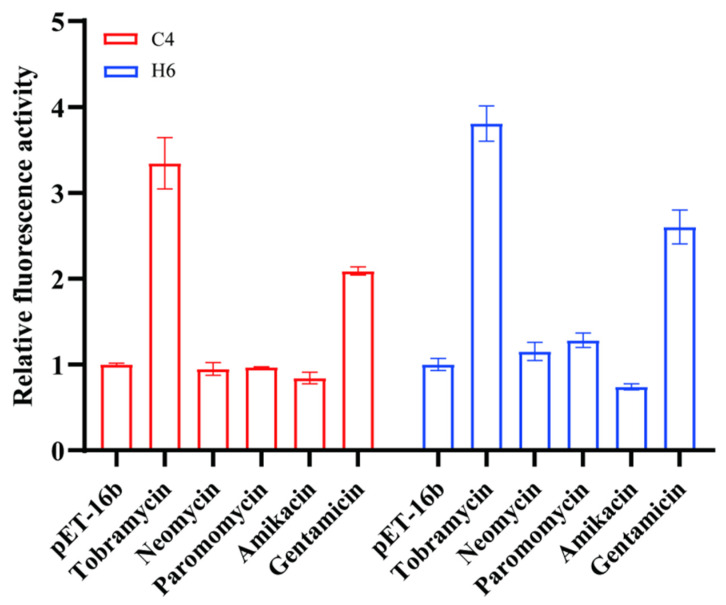
Specificity of tob-HHAz sensors to the aminoglycoside antibiotics. Relative fluorescence activity of tob-HHAz sensors in the presence of aminoglycoside antibiotics (3 µM). Each measurement was performed in triplicate, and the error bars represent the standard deviation of at least three individual experiments.

**Figure 9:**
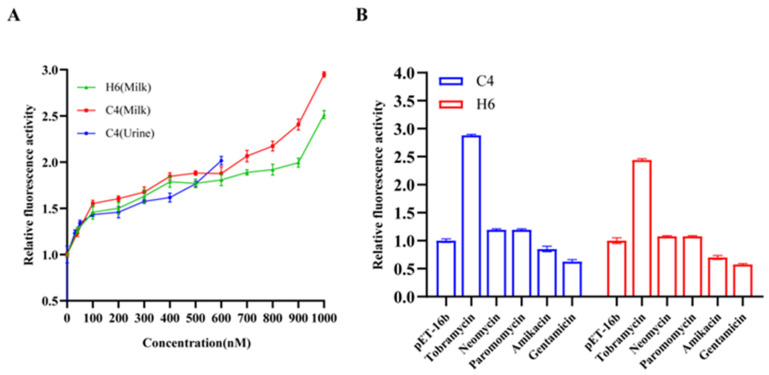
Tobramycin detection in milk and urine: (**A**) qualitative of tobramycin in milk and human urine. Both C4 (red line) and H6 (green line) exhibited high sensor performance with the detection linear range from 0 to 1000 nM, involving a 40 nM minimum detection limit. C4 (blue line) exhibited a linear range from 30 to 650 nM with a LOD of 30 nM in human urine detection. The error bar graph represents the variation between triplicate experiments; (**B**) specificity of the sensor fluorescence assay for tob detection; the concentrations of substances were all 1000 nM. The data were normalized using the fluorescence expression of negative control.

Under 3 µM concentrations, the performance of C4 and H6 sensors was not affected by neomycin, paramycin, and amikacin, showing excellent tobramycin specificity. It is worth noting that both sensors exhibited a 2–3-fold improvement in the relative fluorescence intensity by adding 3 µM of gentamicin (Figure 8). The outcome of the cell vitality test suggests that a higher level of gentamicin has a serious impact on bacterial multiplication (Figure S2D), resulting in a large deviation in the value of the relative fluorescence intensity. Fortunately, we found that a lower gentamicin concentration (less than 1 µM) (Figure S3), which was far more than the threshold value of detection in most real samples, had little effect on bacterial growth; thus, we hypothesized that it might not influence the specificity of these sensors in the real sample test. The results were confirmed in the subsequent experiment (Figure 9B). 

### 3.7. Tobramycin Detection in Milk and Urine

The qualitative and rapid detection of tobramycin in milk samples was performed for verifying the practicability of the assay (Figure 9A). 

C4 and H6 revealed a 2.5–3 fold improvement in dose-dependence fluorescence in the milk sample supplemented with 1000 nM tob. C4 showed better performance than H6, with the perfect detection linear range from 100 to 1000 nM. The signals were detected in milk with 40 nM of tobramycin, which is lower than the maximum residue level (MRL) stipulated by the European Union. 

In the subsequent qualitative detection of human urine, the C4 structure provided a linear range from 30 to 650 nM with a minimum limit of detection (LOD) of 30 nM. This dose-dependent characteristic provides the possibility for ligand concentration assessment in unknown samples. These analysis results with good repetition can meet the test requirement of the standard method. However, in H6, we did not observe a significant improvement in dose-dependence fluorescence in urine as a result of the addition of tob, suggesting that the complex composition of urine has a greater impact on the stability of the sensor for its in vivo detection. 

The selectivity of a sensor is a vital factor for the sensor to be used in real samples due to its complex matrix, possibly containing various components. Considering the same class of aminoglycoside antibiotics, some potential interfering antibiotics involving neomycin, paromomycin, amikacin, and gentamicin were selected for testing. The results are shown in Figure 9B. Only the sample containing tob with a concentration of 1000 nM had a significant fluorescence enhancement, while all others with the same concentrations had no obvious response compared with the blank solution. Although the procedures were carried out with different aptamers individually, C4 and H6 retained their high specificity for tobramycin detection in all the samples.

## 4. Discussion

In recent years, the emergence of synthetic biology has greatly promoted the development of biosensors. Due to its programmable properties, an artificial riboswitch provides a promising method for genetic regulation at different levels. Ribozyme switch can realize the “turn-on” or “turn-off” function of gene expression via splicing rather than a conformational change in RNA. It has a smaller size, modular composition, and high specificity for ligand recognition and therefore can be used as a promising molecular sensing platform. However, there have been no reports of using ribozyme switches as whole-cell sensors to detect antibiotics in real samples. Moreover, due to the complex intracellular environment, RNA aptamers produced via SELEX screening in vitro usually show poor affinity and selectivity in vivo, and only a few aptamers or ribozyme switches screened in vitro have the potential to be used for the detection of small molecule ligands in vivo. The key to the design of an artificial ribozyme switch is the joint sequence between the ligand domain and the expression motif. Here, we designed a library of six random joint sequences and then conducted the high-throughput screening of 96-well plates. The number of clones in the library was eight times the theoretical value to ensure that all the screened clones could be covered. However, with the increasing number of bridge sequences, flow cytometry was essential for effective analysis. In addition, we found that the positive C7 candidates, which failed in terms of their splicing capability in the in vitro experiment, still confirmed the prediction results of the RNA secondary structure. 

Further construction parameters showed that the original stem structure to maintain the exposure of the splicing site was crucial for the efficient cleavage of RNA. Here, the Vienna RNA 2.0 software was used for the dynamic analysis of the RNA secondary structure, the visual analysis of base pairing probability, etc., which provide a useful reference for us to understand the activation efficiency, variable splicing location, and degradation rate of the ribozyme catalytic core motif. The prediction of the secondary structure of artificial allosteric RNA is of great significance for advancing the rational design of ligand-dependent biosensors. 

When applying our sensors to biological samples, we found that in serum samples, due to the complex components in the blood, effective detection was difficult. Although the sensor exhibited ligand-responsive performance for 30/40 nM of tobramycin, it was difficult to use the system for the absolute quantification of the sample in lower concentrations (<100 nM) of tobramycin. We found that the fluorescence expression failed to show a dose–response characteristic, so an important research topic for further applications is the improvement in the sensitivity of the tobramycin sensor with respect to ligand concentrations below 100 nM.

In the future, further research will be carried out to optimize the structure of the sensor and increase its anti-interference capability by improving its sensitivity through further signal amplification design. This strategy enables the use of artificial ribozyme-based biosensors not only for antibiotic detection but also as a generally applicable method for further detection of substances in living cells, thus enabling their wider application in environmental monitoring or medical detection.

## Data Availability

All data, tables, and figures are original. Details on data analysis are available from the corresponding author upon reasonable request.

## Supplementary Materials

The following supporting information can be downloaded at: https://www.mdpi.com/article/10.3390/life13071553/s1, Table S1. List of primer sequences of plasmid construction. Table S2. List of primer sequences of plasmid construction. Table S3. List of primer sequences of in vitro transcription. Figure S1. Establishment of validated concentrations of tobramycin. The validated concentration of tobramycin was determined using an F9 vitality test. The synergy effect of OD600 and fluorescence expression shows the F9 growth state. Figure S2. Establishment of validated concentrations of aminoglycoside antibiotics: (A) neomycin, (B) paramycin, (C) amikacin, and (D) gentamicin. Figure S3. Effects of gentamicin on bacteria multiplied with low concentrations (<1 µM).
