# Supplementary material for: The Establishment of a Tobramycin-Responsive Whole-Cell Micro-Biosensor Based on an Artificial Ribozyme Switch
Source: Life (Basel). 2023 Jul 13;13(7):1553. doi: 10.3390/life13071553 (PMC10381313; doi:10.3390/life13071553)
Supplement: Supplementary file 1 [file life-13-01553-s001.zip › life-2419598-supplementary.pdf]

Supporting Information

Table S1. List of primer sequences of plasmid construction.

Name	Sequences	Template
pET- eGFP	P1 : CCGCATATGATGGTGAGCAAGGGCGAG<br>P2 : GCGGATCCGCTTCTTGTACAGCTCGTCCATG	pET-16b
pET- eGFP- HHR	P1:<br>GATAACAATTCCCCTTCTCCTTCGGTACATCCAGCTGATGAGTCCCAAATAGGACGAAACGCGCTTCGGTGCGTCCTGGATTCCACGAAGGAGATATACCA<br>P2:<br>TGGTATATCTCCTTCGTGGAATCCAGGACGCACCGAAGCGCGTTTCGTCCTATTTGGGACTCATCAGCTGGATGTACCGAAGGAGAAGGGGAATTGTTATC<br>P1 - 1 : TTCCACGAAGGAGATATACCATGGGCA<br>P2 - 1 : AGGAGAAGGGGAATTGTTATCCGCTCA	pET- eGFP
pET- eGFP- HHR- TOB -A1	P1 : TAAACCTCGTGCC*NNN*TTTCGTCCTATTTGGGACT<br>P2 : GCTACACTCCTGCC*NNN*TCCTGGATTCCACGAAGG	pET- eGFP- HHR
pET- e EGFP- HHR- TOB -A2	P1 : CCTAGTC*NNN*TTTCGTCCTATTTGGGAC<br>P2 : CACTAGTC*NNN*TCCTGGATTCCACGAA	pET- eGFP- HHR
pET- eGFP- HHR- TOB -A3	P1 : TAAACCA*NNN*TTTCGTCCTATTTGGGACT<br>P2 : GGTAATG*NNN*TCCTGGATTCCACGAAG	pET- eGFP- HHR
random primer(for screening of Anti- RBS)	P1 : GATAACAATTCCCCTT*NNNNN*TCGGTACATC<br>P2 : TGGTATATCTCCTTCGTGGAATCCAGG	pET- eGFP- HHR

The single underline represents the restriction enzyme sites of *Nde I* and *BamH I.* The double underline represents the complementary regions of the primer and the template. The italics N represent the random DNA linkage sequence between the HHR ribozyme and the aptamer (N=A/T/C/G).

Table S2. List of primer sequences of plasmid construction.

Name	Sequence
H3(HHR variant)	CATGGTATATCTCCTTCGTGGAATCCAGGACGCACCGAAGCGCGTTTCGTCCTATTTGGGACTCATCAGCTGGATGTACCGATCAAGA
F9(HHR variant)	CATGGTATATCTCCTTCGTGGAATCCAGGACGCACCGAAGCGCGTTTCGTCCTATTTGGGACTCATCAGCTGGATGTACCGAAACGAA
D6(HHR variant)	CATGGTATATCTCCTTCGTGGAATCCAGGACGCACCGAAGCGCGTTTCGTCCTATTTGGGACTCATCAGCTGGATGTACCGACGTCAA
C4	CATGGTATATCTCCTTCGTGGAATCCAGGA*ATG*GGCAGGAGTGTAGCTAAACCTCGTGCC*CGA*TTTCGTCCTATTTGGGACTCATCAGCTGGATGTACCGATCAAGA
C7	CATGGTATATCTCCTTCGTGGAATCCAGGA*CGC*GACTAGTGCCTAGTC*GGT*TTTCGTCCTATTTGGGACTCATCAGCTGGATGTACCGATCAAGA
D7	CATGGTATATCTCCTTCGTGGAATCCAGGA*ATC*GACTAGTGCCTAGTC*CCG*TTTCGTCCTATTTGGGACTCATCAGCTGGATGTACCGATCAAGA
H6	CATGGTATATCTCCTTCGTGGAATCCAGGA*TGG*GACTAGTGCCTAGTC*GTC*TTTCGTCCTATTTGGGACTCATCAGCTGGATGTACCGATCAAGA

The red nucleotides indicate the mutation sequences. The italics represent the linkage sequences between the HHR ribozyme and the aptamer.

Table S3. List of primer sequences of in vitro transcription.

Name	Sequences	Template
SL-F	ATATGGCCGCTGCTGTGATGATG	H3 F9 C4 D7 H6 and mutants
SL-R	TAATACGACTCACTATAGGGGAATTGTGAGC	
C4-TOB-SS-1-F	GAATGGGCAGGAGTGTAGCTAAAC	C4
D7-TOB-SS-1-F	GACGCGACTAGTGCCTAGTCGGTTTTC	D7 H6

The double underline represents the complementary regions of the primer and template.

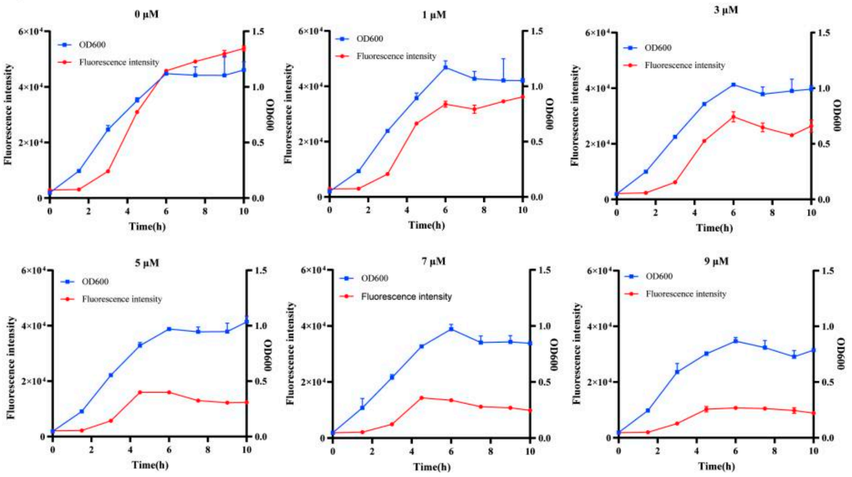

Figure S1. Establishment of validated concentrations of tobramycin. The validated concentrations of tobramycin was determined using an F9 vitality test. The synergy effect of OD600 and fluorescence expression shows the F9 growth state.

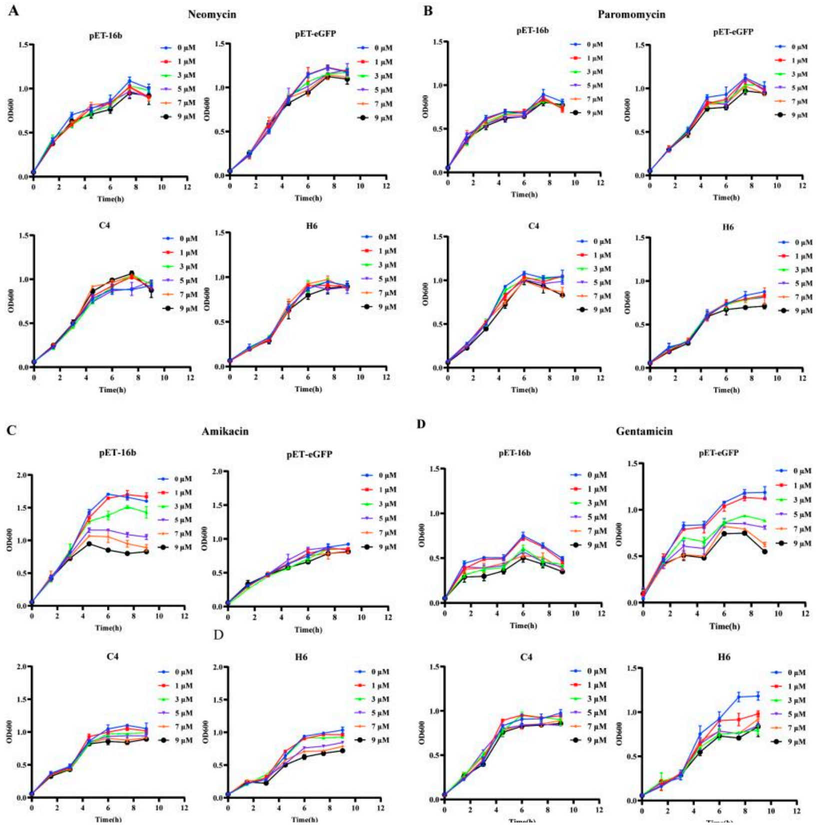

Figure S2. Establishment of validated concentrations of aminoglycoside antibiotics: **(A)** neomycin, **(B)** paramycin, **(C)** amikacin, and **(D)** gentamicin.

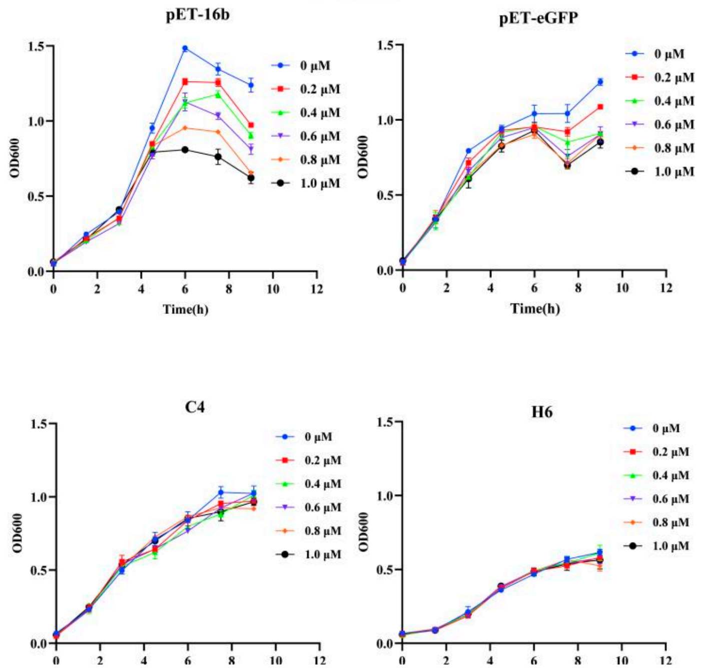

Figure S3. Effects of gentamicin on bacteria multiplied with low concentrations (<1 μM).
